# The Comparative Evaluation of the Anesthetic Efficacy of 4% Articaine With 1:100,000 Adrenaline and 0.75% Ropivacaine for Inferior Alveolar Nerve Block in the Extraction of Impacted Lower Third Molar

**DOI:** 10.7759/cureus.29639

**Published:** 2022-09-27

**Authors:** Nupoor Deshpande, Anendd Jadhav, Nitin D Bhola, Manan Gupta

**Affiliations:** 1 Oral and Maxillofacial Surgery, Nishant Dental Care, Raipur, IND; 2 Oral and Maxillofacial Surgery, Sharad Pawar Dental College and Hospital, Datta Meghe Institute of Medical Sciences, Wardha, IND

**Keywords:** local anesthetics, ropivacaine, third molar, articaine, analgesia

## Abstract

Introduction

Postoperative pain management is a major concern in lower third molar surgery. Various local anesthetic agents are studied for the same. Articaine and ropivacaine are recently studied for pain control intra- and postoperatively in minor oral surgical procedure. However, there is sparse literature that compares these two agents. Therefore, the present study was designed to compare 4% articaine with 1:100,000 adrenaline and 0.75% ropivacaine for inferior alveolar nerve block in lower impacted third molar surgery.

Materials and method

A prospective, randomized controlled, split-mouth, double-blind study was performed. A total of 60 healthy patients requiring extraction of lower impacted third molar with similar difficulty index were included in the study. Patients were administered 4% articaine with 1:100,000 adrenaline and 0.75% ropivacaine on either side by random allocation. The procedure was performed at an interval of 14 days. Parameters assessed were time of onset of anesthesia, profoundness of anesthesia, hemodynamic parameters (heart rate and blood pressure), duration of soft tissue anesthesia, duration of postoperative analgesia, and postoperative symptom severity (PoSSe) scale.

Results

The time of onset of articaine was faster (69.20 + 20.13 seconds) compared to ropivacaine (104.06 + 17.66 seconds). No significant difference was observed in hemodynamic parameters. There was significant difference in duration of soft tissue anesthesia, postoperative analgesia, and PoSSe scale between the two groups.

Conclusion

Within the limitations of the study, 0.75% ropivacaine was effective in providing prolonged soft tissue anesthesia, postoperative analgesia, and better PoSSe scale with hemodynamic stability compared to 4% articaine.

## Introduction

Lower third molar surgery is a frequently encountered procedure in dental practice, which can be associated with severe postoperative pain, edema, and trismus caused by surgical trauma leading to a cascade of inflammatory events and the release of inflammatory mediators. All of these lead to patient discomfort and adversely affect the quality of life, which mandates effective postoperative pain control [1]. The use of effective local anesthetic agents prolongs the pain-free postoperative period resulting in better psychological outcomes of the patient.

Articaine consists of lipophilic thiophene ring instead of the usual benzene ring, which increases its lipid solubility and gives the molecule excellent property of diffusion through hard and soft tissues. The clinical advantage of articaine includes faster metabolism in blood plasma due to the ester group and duration of anesthesia due to higher protein-binding property, which can only be surpassed by long-acting local anesthetic agents such as bupivacaine and ropivacaine. It also exhibits lower cardiovascular system (CVS) and central nervous system (CNS) toxicity [2].

Ropivacaine is a long-acting local anesthetic agent introduced for clinical use in 1996 with similar structure to bupivacaine. Due to its properties such as longer duration of action and low toxicity, it has been successfully used in impacted lower third molar surgeries [3].

Like all other local anesthetic agents, articaine shows vasodilatory effect. Braun suggested the addition of vasoconstrictor that prolongs the duration of action [4]. However, the use of vasoconstrictor such as adrenaline in higher doses introduces the risk of systemic toxicity [5]. In contrast, ropivacaine is the only anesthetic agent that shows biphasic vascular effect. At low concentration (0.5%), it shows vasoconstriction and vasodilation at high concentration (1%). So, the use of ropivacaine in low concentration eliminates the need of vasoconstrictor, thereby reducing the toxicity [3].

Articaine and ropivacaine are two widely studied local anesthetic agents for pain control in minor oral surgical procedure and have proved to provide patient comfort intra- and postoperatively [6,7]. However, no studies compare the effect of these two drugs when used for minor oral surgical procedure. Therefore, the aim of this study was to compare the anesthetic efficacy of 4% articaine with 1:100,000 adrenaline and 0.75% ropivacaine for inferior alveolar nerve block in impacted lower third molar surgery.

## Materials and methods

This prospective, double-blind, split-mouth, randomized clinical study was performed on 60 individuals in the outpatient department of oral and maxillofacial surgery, Sharad Pawar Dental College, Sawangi, from September 2015 to August 2018. The study was performed in accordance with the Helsinki declaration and its later amendments or comparable ethical standards and institutional ethical guidelines prescribed by the Central Ethics Committee on Human Research (CECHR) (reference number DMIMS (DU)/IEC/ 2015-16/1668).

A total of 60 healthy patients (American Society of Anesthesiologists {ASA} I) were selected by convenience sampling between 20 and 40 years of age presenting with bilateral impacted lower third molars with similar Pederson difficulty index of mesioangular; class 2 and position B according to Pell and Gregory classification were included in the study. The formula used to calculate the sample size required for this study at 95% confidence interval and 80% power of significance was N = (Zɑ / 2 + Zβ) 2 × 2 × σ2 / d2. The calculated sample size for this study was 25 for each group; taking into account the loss of follow-up, a sample size of 30 for each group was devised.

Patients with a history of any systemic diseases, lactating or pregnant women, patients on antidepressants, smokers, infection on the site of intervention, and allergy to local anesthetic agents or anti-inflammatory agents were excluded from the study. Also, the presence of radiographic evidence of inferior alveolar canal involvement was excluded.

A written informed consent was taken from each patient before the procedure was initiated. Thorough clinical and radiographic examination was done, and each patient was subjected to blood investigations such as hemoglobin, bleeding time, and clotting time. Patients were explained about the procedure and numeric visual analog scale (VAS) [8]. Baseline heart rate and blood pressure were recorded.

The implementation of the study was based on split-mouth study model where patients undergoing impacted lower third molar extraction were administered 2 ml 0.75% ropivacaine (Ropin 0.75%, Neon Laboratories Ltd, India) as inferior alveolar nerve block on one side and 2 ml 4% articaine with 1:100,000 adrenaline (Septanest, Septodont Healthcare India Pvt Ltd, India) on the contralateral side. The extraction on both sides was done at an interval of 14 days [9]. Both solutions were loaded in identical transparent syringes before administration. The randomization of the side was done prior to conducting the study. A member was recruited in the study in the capacity of an independent observer for recording pain and discomfort during the administration of anesthesia and dental extraction. This member was not further involved in statistical analysis to avoid bias. Both the participant and the observer were blinded to the drug being administered.

One appointed surgeon performed inferior alveolar nerve block and surgical lower third molar extraction for every patient. Ward I/modified ward's incision was given to all patients. A sharp periosteal elevator was used to reflect the full-thickness mucoperiosteal flap in the subperiosteal plane. Once the mucoperiosteal flap was reflected, the tooth was exposed, and the bone overlying the crown of the impacted third molar was removed by round bur (number 702) around the tooth, and the bone guttering was done under copious irrigation with 0.9% normal saline. Following the removal of the tooth, the extraction socket was packed with the help of cotton gauze to achieve initial hemostasis and was examined thoroughly for the existence of any foreign particle. Any sharp bony margins around the socket were trimmed with the help of a round bur and smoothened using a bone file. Any lacerated margins of the flap were removed with sharp scissors. The surgical site was closed with interrupted sutures using 3-0 Mersilk. Sutures were removed after seven days.

Post-recruitment exclusion criteria included patient reporting pain >3 on visual analog scale during the surgery and needing re-anesthesia and any postoperative neurosensory deficit. Postoperatively, patients were prescribed capsule amoxicillin 500 mg three times a day (tid) and tablet ibuprofen 400 mg tid for five days along with antibacterial mouth wash. Patients were given the VAS chart and asked to consume analgesic only when pain exceeded >3 on VAS. The consumption of analgesic was considered the end point of the study.

The patient was asked to wait in the clinic for about two hours to assess the study parameters. Parameters assessed were as follows: a) time of onset of anesthesia, defined as the time of injection to the time when the effect of anesthesia was first reported and objectively assessed with the use of blunt probe on the gingiva adjacent to the tooth; b) profoundness of anesthesia, defined as the pain experienced by the patient during various events of the procedure (probing, flap reflection, bone guttering, and tooth elevation/suturing) and recorded on VAS [6]; c) hemodynamic changes, in which heart rate and blood pressure (systolic blood pressure {SBP} and diastolic blood pressure {DBP}) were recorded at 10, 30, 60, and 120 minutes following injection during the procedure; d) duration of soft tissue anesthesia, defined as the time of the onset of anesthesia to the time of the cessation of numbness of the lip and tongue; e) duration of postoperative analgesia, defined as the time of the completion of the procedure to the time of the consumption of analgesic; and f) PoSSe scale [10,11], which is the postoperative symptom severity scale assessed under seven subscales (eating, speech, sensation, appearance, pain, sickness, and interference with daily activities) and was completed by the patient on seventh day of surgery.

**Figure 1:**
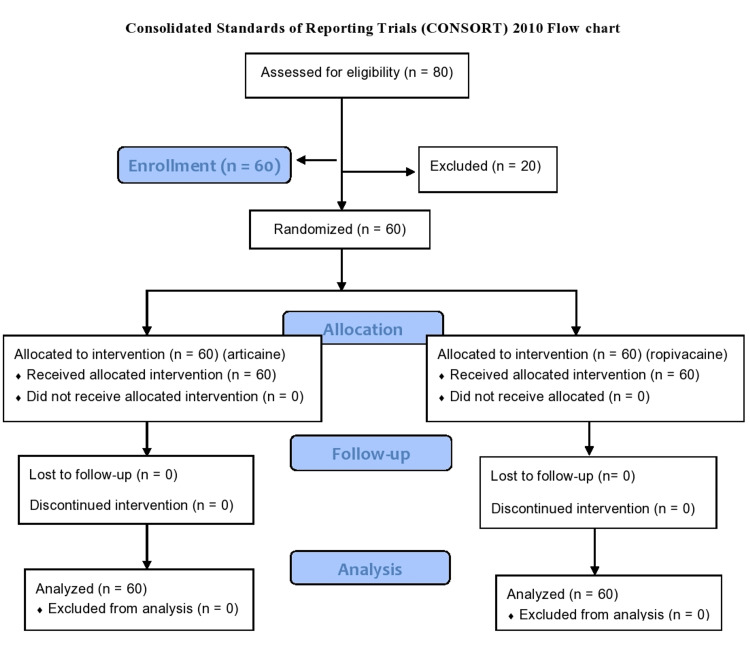
CONSORT flow chart CONSORT: Consolidated Standards of Reporting Trials

All the findings were recorded in Microsoft Excel (Microsoft® Corp., Redmond, WA) sheet and were statistically analyzed. The Statistical Package for Social Sciences (SPSS) version 21.0 (IBM Corp., Chicago, IL) was used. The significant differences between both groups were assessed using Student's t test for unpaired samples and chi-square test. Statistical significance was established at p < 0.05. Consolidated Standards of Reporting Trials (CONSORT) 2010 [12] flow chart is shown in Figure 1.

## Results

The study consisted of 33 males and 27 females with a mean age of 27.27 ± 6.14 years (ranging from 23 to 35 years). The time of onset of anesthesia with articaine was 69.20 ± 20.13 seconds and with ropivacaine was 104.06 ± 17.66 seconds; the difference was found to be statistically significant (p < 0.001). There was no significant difference in pain intensity as measured on VAS during various events of surgery (probing, flap reflection, bone guttering, and tooth elevation/suturing). The data showed a significant difference in duration of soft tissue anesthesia and duration of analgesia between the two groups (p < 0.001). In the PoSSe scale when compared between two groups, the difference was found to be statistically significant (p < 0.001) (Table 1). 

**Table 1:** Mean observation of various parameters made preoperatively, intraoperatively, and postoperatively VAS: visual analog scale; PoSSe: postoperative symptom severity; NS: not significant *Statistically significant difference

Variables	Articaine	Ropivacaine	P value
Time of onset of anesthesia (seconds)	69.20 ± 20.13	104.06 ± 17.66	0.000*
Pain intensity (VAS)			
Probing	0	0	-
Flap reflection	0	0	-
Bone guttering	0.20 ± 0.48	0.13 ± 0.43	0.577 (NS)
Tooth elevation/suturing	0.86 ± 0.73	0.76 ± 0.77	0.609 (NS)
Duration of soft tissue anesthesia (hours)	1.90 ± 0.58	5.89 ± 0.79	0.000*
Duration of analgesia (hours)	2.11 ± 0.53	6.99 ± 1.34	0.000*
Full PoSSe scale	22.86 ± 6.61	15.26 ± 5.55	0.000*

There was transient rise in heart rate at an interval of 10 minutes on the administration of articaine, and gradually, this reduced to normal range at 120 minutes. The difference in heart rate between the two groups was statistically insignificant (p = 0.473, p = 0.497, p = 0.968, and p = 0.773). The blood pressure (SBP and DBP) between the two groups at every interval was statistically insignificant (p < 0.05) (Table 2).

**Table 2:** Mean changes in hemodynamic parameters between the two groups NS: not significant

Variables	Articaine	Ropivacaine	P value
Heart rate (beats/minute) (baseline)	77.13 ± 6.67	78.73 ± 8.14	0.409 (NS)
10 minutes	81.33 ± 5.36	80.13 ± 7.35	0.473 (NS)
30 minutes	80.93 ± 5.16	79.86 ± 6.80	0.497 (NS)
60 minutes	78.80 ± 5.42	78.73 ± 7.17	0.968 (NS)
120 minutes	76.86 ± 5.55	76.00 ± 15.42	0.773 (NS)
Blood pressure (mmHg)			
Baseline systolic	116.80 ± 8.31	115.06 ± 9.75	0.462 (NS)
Baseline diastolic	75.20 ± 9.60	75.80 ± 6.54	0.778 (NS)
10 minutes			
Systolic	120 ­± 7.08	117.40 ± 8.96	0.218 (NS)
Diastolic	78.06 ± 8.31	77 ± 5.47	0.560 (NS)
30 minutes			
Systolic	119.93 ± 6.95	116.93 ± 6.36	0.087 (NS)
Diastolic	77.20 ± 6.79	75.80 ± 5.41	0.381 (NS)
60 minutes			
Systolic	117.86 ± 6.47	117 ± 8.08	0.648 (NS)
Diastolic	75.40 ± 7.18	76.60 ± 4.95	0.455 (NS)
120 minutes			
Systolic	116.93 ± 6.76	115.93 ± 8.19	0.608 (NS)
Diastolic	74.93 ± 7.66	75.20 ± 5.05	0.874 (NS)

## Discussion

The advent of various local anesthetic agents has led to effective prevention and management of pain in minor oral surgical procedures. Long-acting, safe, and efficient local anesthetic agents are required to overcome the postoperative discomfort of the patient. Articaine and ropivacaine are two such drugs that are studied widely in impacted lower third molar surgeries. Articaine, since its discovery in 1969, is known for its high diffusion rate and lipid solubility due to the presence of thiophene ring [2]. Ropivacaine, a long-acting agent, provides prolonged duration of action, thereby increasing the postoperative pain-free period. Both drugs exhibit low CVS and CNS toxicity [3]. Although there are studies of the use of articaine and ropivacaine in minor oral surgical procedures, direct comparison of these two agents is limited. Hence, the present study was aimed to compare the efficacy and safety of 4% articaine with 1:100,000 adrenaline and 0.75% ropivacaine for inferior alveolar nerve block for lower impacted third molar surgery.

The present double-blind, split-mouth, prospective study included 60 patients with bilaterally lower third molars with mean age of 27.27 ± 6.14 years (ranging from 23 to 35 years). All the extractions were performed in a standardized, controlled, and patient-friendly environment by the same surgeon at an interval of 14 days. The results of the study show that the mean time of onset of anesthesia for articaine (69.20 ± 20.13 seconds) was faster compared to ropivacaine (104.06 ± 17.66 seconds) with a statistically significant difference (p < 0.001). The latency of the drug depends on the intrinsic property of the drug and dissociation constant (pKa value). Latency is directly proportional to pKa of a drug; therefore, the smaller the pKa, the shorter the latency period [13]. Hence, faster onset of articaine may be attributable to its pKa value of 7.8, which is close to the body pH and therefore enables the rapid diffusion into tissue, whereas ropivacaine has a pKa of 8.1, thereby increasing its latency period [14]. No studies in literature show direct comparison of these two drugs for inferior alveolar nerve block in minor oral surgery. However, similar result with the time of onset of articaine was found in the studies by Saralaya et al. (2019) [6], Jain and John (2016) [15], and Mittal et al. (2018) [16]. Also, similar results were found for ropivacaine [3,17,18].

The profoundness of anesthesia was measured by assessing pain intensity on visual analog scale (VAS) for various events such as probing, flap reflection, bone guttering, and tooth elevation/suturing. None of the patients had pain during any events of the procedure. Similar results were reported by Kambalimath et al. (2013) [13] and Jain and John (2016) [15] for articaine and Budharapu et al. (2015) [3] for ropivacaine when these drugs were compared to lignocaine.

Hemodynamic parameters such heart rate and blood pressure (SBP and DBP) were recorded at various intervals of 10, 30, 60, and 120 minutes following the administration of local anesthetic agents. Changes in hemodynamic parameters are subjected to the addition of vasoconstrictor and adrenaline that delays the absorption of drug in the cardiovascular system, increases the duration of action, and reduces the risk of toxicity. It causes transient increase in heart rate and blood pressure and also increase in heart rate and blood pressure suffer variation during stressful situations [19,20].

Almost all the local anesthetic agents, including articaine, are vasodilators requiring the addition of vasoconstrictor that may cause change in hemodynamic parameters. However, ropivacaine has a property of vasoconstriction at low concentration. In the present study, there was transient increase in heart rate and blood pressure in both groups at 10 minutes, which returned to the baseline values on subsequent measurements. In the rest of all intervals, the difference in heart rate and blood pressure between the two groups was found to be insignificant (p > 0.05). Kambalimath et al. (2013) [13], Budharapu et al. (2015) [3], and Reddy et al. (2019) [18] reported similar results with articaine and ropivacaine when compared to lignocaine, respectively.

Duration of soft tissue anesthesia depends on protein binding of the drug. Articaine has a high protein binding (94%), which can only be surpassed by long-acting local anesthetic agents such as ropivacaine [14]. When compared between articaine and ropivacaine, the study results showed longer duration of anesthesia with ropivacaine (5.89 + 0.79 hours) than articaine (1.90 + 0.58 hours) with a significant difference (p < 0.05).The longer duration of action of ropivacaine may be due to its inherent vasoconstriction property. Similar results were observed in previous studies. Krzemiński et al. (2011) [21] and Bansal et al. (2018) [22] reported longer duration of anesthesia with ropivacaine. Also, the duration of anesthesia for articaine is in accordance to studies performed by Saralaya et al. (2019) [6], Kambalimath et al. (2013) [13], and Jain and John (2016) [15]. Though prolonged anesthesia provides pain-free postoperative period, it can also be associated with lower lip injury and patient discomfort.

The duration of post-operative analgesia with articaine and ropivacaine was 2.11 + 0.53 hours and 6.99 + 1.34 hours, respectively, with a significant difference (p < 0.001). Patients were asked to consume rescue analgesics only when pain on VAS was >3. Patients were given postoperative symptom severity scale, which consisted of seven subscales (eating, speech, sensation, pain, appearance, and interference with daily activities). The full PoSSe score with articaine and ropivacaine showed significant difference (p < 0.05) [11]. These results suggest that ropivacaine provides better postoperative pain-free period and less discomfort owing to its high potency and longer duration of action.

This study did not take into consideration the plasma levels of both drugs, which would have provided the detailed effect on the cardiovascular system. The safety of drugs in patients with compromised cardiovascular system was not evaluated. Also, multi-centric trials with larger sample size are needed to support the result of this study.

## Conclusions

Articaine and ropivacaine are two agents that are used widely these days, but there is a lack of literature on the direct comparison of the two drugs. Therefore, this study was designed to compare the effectiveness between the two agents. Within the limitations of this study, 0.75% ropivacaine shows superior properties than 4% articaine with 1:100,000 adrenaline in terms of longer duration of action and prolonged postoperative analgesia. However, both articaine and ropivacaine are safe with respect to hemodynamic parameters.
